# Analysis of bioavailable toluene by using recombinant luminescent bacterial biosensors with different promoters

**DOI:** 10.1186/s13036-020-00254-1

**Published:** 2021-01-06

**Authors:** Guey-Horng Wang, Teh-Hua Tsai, Chun-Chi Kui, Chiu-Yu Cheng, Tzu-Ling Huang, Ying-Chien Chung

**Affiliations:** 1 Research Center of Natural Cosmeceuticals Engineering, Xiamen Medical College, Xiamen, 361008 China; 2 Department of Chemical Engineering and Biotechnology, National Taipei University of Technology, Taipei, Taiwan; 3 Department of Biological Science and Technology, China University of Science and Technology, Taipei, 11581 Taiwan

**Keywords:** Biosensor, Groundwater, Promoter, Toluene

## Abstract

In this study, we constructed recombinant luminescent *Escherichia coli* with T7, T3, and SP6 promoters inserted between *tol* and *lux* genes as toluene biosensors and evaluated their sensitivity, selectivity, and specificity for measuring bioavailable toluene in groundwater and river water. The luminescence intensity of each biosensor depended on temperature, incubation time, ionic strength, and concentrations of toluene and coexisting organic compounds. Toluene induced the highest luminescence intensity in recombinant *lux*-expressing *E. coli* with the T7 promoter [T7-*lux-E. coli*, limit of detection (LOD) = 0.05 μM], followed by that in *E. coli* with the T3 promoter (T3-*lux-E. coli*, LOD = 0.2 μM) and SP6 promoter (SP6-*lux-E. coli*, LOD = 0.5 μM). Luminescence may have been synergistically or antagonistically affected by coexisting organic compounds other than toluene; nevertheless, low concentrations of benzoate and toluene analogs had no such effect. In reproducibility experiments, the biosensors had low relative standard deviation (4.3–5.8%). SP6-*lux-E. coli* demonstrated high adaptability to environmental interference. T7-*lux-E. coli* biosensor—with low LOD, wide measurement range (0.05–500 μM), and acceptable deviation (− 14.3 to 9.1%)—is an efficient toluene biosensor. This is the first study evaluating recombinant *lux E. coli* with different promoters for their potential application in toluene measurement in actual water bodies.

## Introduction

The large-scale consumption of petroleum-derived fuels has led to groundwater and soil contamination through their leakage from fuel tanks and pipelines. Because of its moderate solubility in water and toxicity, toluene is a petrochemical contaminant of particular concern [1]. Even at low concentrations, toluene can be carcinogenic, can exhibit mutagenic properties, and can damage the kidney, liver, and central nervous system [2]. In Taiwan, environmental agencies have set acceptable limits for toluene in drinking water and groundwater at considerably low levels (7.6–10.9 μM) [3, 4]. In addition, toluene measurement is paramount for the monitoring and clean-up of contaminated groundwater and surface water. Thus, the need for sensitive toluene detection is high, but its design is challenging. In particular, toluene is found in various water bodies, including rivers, as well as coastal water and groundwater; even drinking water contains toluene at trace concentrations (μM) [1].

Conventional analytical techniques, such as gas chromatography (GC) and high-performance liquid chromatography, are sensitive and reliable for toluene detection but are time-consuming, expensive, and laboratory-bound, and they require large equipment and specialized training [5, 6]. By contrast, biological methods can be useful alternatives for organics detection because they are low cost, easy to use, portable, small, and highly specific and can detect bioavailability [7–9]. Of the biological methods, biosensors are suitable for application as environmental sensors, even for on-field measurements.

Over the last 20 years, biosensors have been developed and are widely used as simple and practical approaches for the sensitive and specific detection of various compounds, including organic compounds (pesticides and chlorophenol), heavy metals (mercury, zinc, and cadmium), and some inorganic compounds [9–11]. Whole-cell biosensors rely on gene expression analysis: transcriptional fusions between a promoter and a reporter gene are created, and the extent of reporter gene expression is used to indicate the pollutant concentration [12]. Several engineered biosensors have specifically discriminated between alkyl-substituted benzene derivatives in water samples [13].

A biosensor of this type can be genetically engineered by placing a reporter gene, such as *lacZ*, *gfp*, *luc*, or *lux*, under the control of a transcriptional activator [11, 14]. Under appropriate conditions (e.g., in the presence of specific pollutant), the biosensor can produce a detectable signal (color or luminescence) that is directly correlated to the pollutant concentration [12, 15]. This property can aid in directly correlating the toluene concentration with the reporter enzyme activity. Various biosensors for benzene, toluene, ethylbenzene, and xylene detection have been developed on the basis of the *tol* plasmid of *Pseudomonas putida* mt-2 [16, 17]. In particular, bioluminescence is highly applicable as a reporter for pollutant detection because its instrumentation is sensitive for detecting light production [18]. However, *Escherichia coli* cells harboring this plasmid often express various response levels when constructed with different reporter genes or promoters that can lead to a range of linear measurement ranges and limits of detection (LODs) [19]. For instance, among induction-based biosensors, *luc-*, *lux*-, and aequorin-based biosensors have the LODs of 11, 7.5, and 1 μM, respectively [20–22]. Of the reporter genes, *lux* has acceptable sensitivity for signal production [18]. Measurement of toluene at very low concentration levels is a main goal of current environmental research; therefore, for the practical application of these biosensors, efforts toward overcoming the aforementioned limitations are warranted [23]. Rational selection of a suitable promoter or reporter gene is essential for increasing the sensitivity, signal intensity, and response speed of whole-cell biosensors.

SP6, T3, and T7 promoters, which are widely used for in vitro transcription, have similar but distinct promoter specificities [24]. They are classified as strong or weak promoters according to their RNA polymerase affinities. T7 is a strong promoter that maintains gene expression tuned to the highest level, thus potentially producing high signal intensity [25]. By contrast, weaker promoter (T3 and SP6) may adapt environmental variation, which produces different signal characteristics [26]. Thus, the linear measurement ranges and LODs of whole-cell biosensors would be expanded or improved if recombinant luminescent bacteria with suitable promoters are constructed.

In this study, we applied this strategy to construct recombinant *E. coli* strains carrying the *tol* plasmid from *P. putida* and including various promoters (T7, T3, or SP6) controlling *lux* expression. By optimizing the promoter and regulating the *lux* expression level in *E. coli*, the recombinant luminescent biosensors could detect bioavailable toluene under different environmental conditions.

## Results and discussion

### Comparison of time-dependent induction of our three recombinant luminescent *E. coli* strains with toluene

**Fig. 1:**
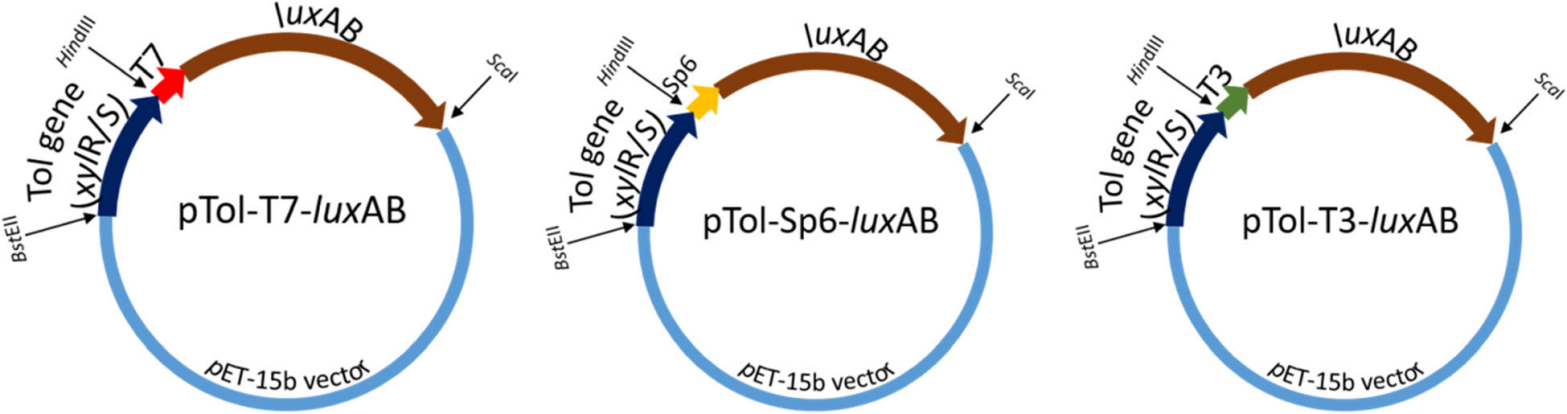
Construction of pTOL-T3-*lux*, pTOL-SP6-*lux* and pTOL-T7-*lux*

**Fig. 2:**
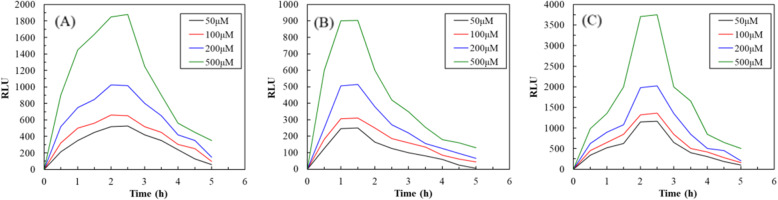
Comparison of time-dependent induction of luminescence from (**a**) T3-*lux-E. coli*, (**b**) SP6-*lux-E. coli*, and (**c**) T7-*lux-E. coli*; initial cell concentration: 5 × 10^7^ cfu/mL, culture media: TMM with different toluene concentration, operational conditions: 37 °C and 200 rpm

Figure 1 illustrates the construction of the three recombinant plasmids. According to the preliminary experiment, the logarithmic growth phases of the three recombinant *E. coli* strains occurred from 6 to 15 h of incubation, and the relationship between OD of bacterial growth and RLU (Relative Light Unit) emitted from the three recombinant *E. coli* strains was linear from 8 to 14 h of incubation. Accordingly, the inoculation time of the three recombinant *E. coli* strains for the subsequent experiment was set as 12 h after incubation. Figure 2 presents a comparison of the time-dependent induction of luminescence emitted from T3-*lux-E. coli*, SP6-*lux-E. coli*, and T7-*lux-E. coli* caused by different toluene concentrations. As shown in Fig. 2, the induction of luminescence caused by different toluene concentrations occurred time-dependently, regardless of the promoter type. The luminescence intensity continuously increased, leveled off, and then began to decrease considerably during incubation, all potentially due to the biochemical nature of the reporter gene *lux* [27].

The results demonstrated that luminescence was stable and the greatest at 2–2.5 h after incubation for T3-*lux-E. coli* and T7-*lux-E. coli* or 1–1.5 h after incubation for SP6-*lux-E. coli*. The time was equal to or shorter than that previously reported for the *lux*-based bioluminescent bioreporter *P. putida* TVA8 (2 h) and luminescence bacterial biosensors without the T7 promoter (3 h) for toluene measurement [6, 21]. Therefore, on average, 20-min consecutive measurements were recorded when T3-*lux-E. coli* and T7-*lux-E. coli* were cultured for 2 h and when SP6-*lux-E. coli* was cultured for 1 h. The maximum average luminescence induced by 200 μM toluene for T3-*lux-E. coli*, SP6-*lux-E. coli*, and T7-*lux-E. coli* was 1020 ± 20, 510 ± 10, and 2120 ± 60 RLU, respectively. Moreover, at the same toluene concentration, the signal intensity of luminescence decreased as follows: T7-*lux-E. coli* > T3-*lux-E. coli* > SP6-*lux-E. coli*. However, SP6-*lux-E. coli* had the shortest stable period for luminescence induction. Previous research demonstrated an increase in bioluminescence emission by fusing the T7 promoter to control expression of the *lux* operon [28].

### Effects of culture conditions on luminescence

**Fig. 3:**
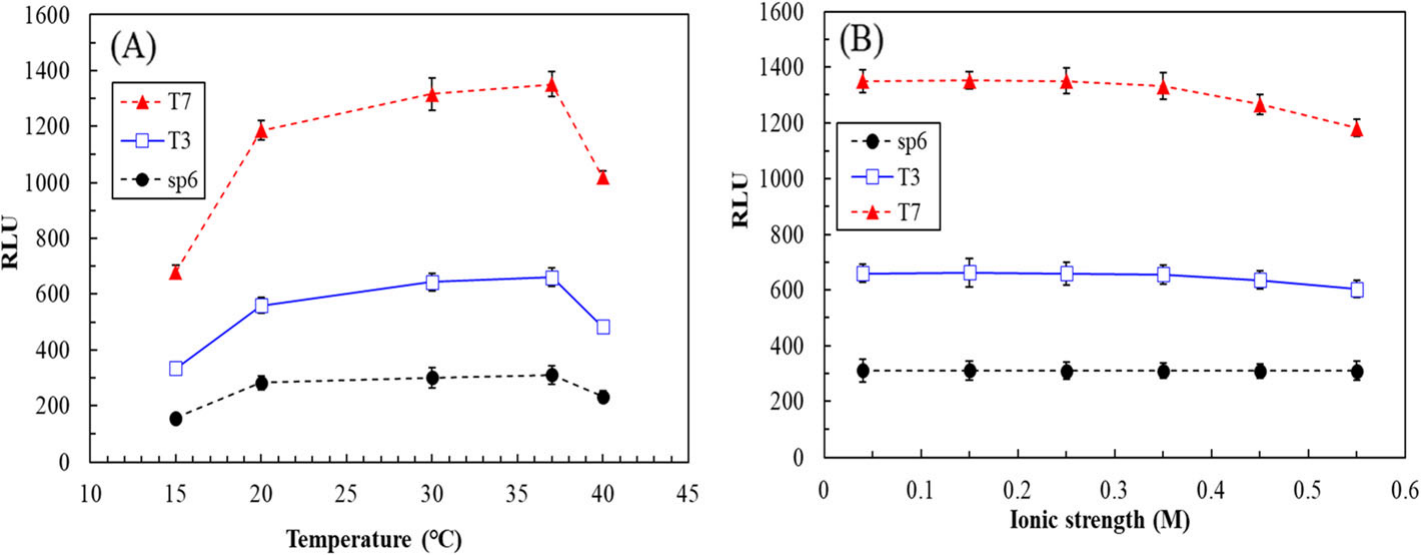
**a** Effects of incubation temperature on luminescence of T7-*lux*-*E. coli* induced by 100 μM toluene for 2 h. **b** Effects of ionic strength on the luminescence of T3-*lux-E. coli*, SP6-*lux-E. coli* and T7-*lux-E. coli* induced by 100 μM toluene for 2 h (T3-*lux-E. coli* and T7-*lux-E. coli*) or 1 h (SP6-*lux-E. coli*)

The effects of incubation temperature and ionic strength on the induction of luminescence biosensors for toluene were evaluated according to practical considerations. Figure 3a illustrates the effects of incubation temperature on the luminescence induced by 100 μM toluene for T7-*lux-E. coli*. The experimental results demonstrated the optimal temperature range of luminescence for T7-*lux-E. coli* to be 30–37 °C, with nonsignificant differences (*p >* 0.05). Similar results were observed for T3-*lux-E. coli* and SP6-*lux-E. coli*. Moreover, luminescences of the three recombinant *E. coli* strains at 20 and 40 °C were 12.1–15.3% and 24.4–26.8% lower than those at 37 °C, respectively. The effect of high temperature on the luminescence of the recombinant *E. coli* strain was more noticeable, a result attributable to the physiological characteristics of the *E. coli* [29]. Thus, subsequent experiments were performed at 37 °C for all three recombinant *E. coli* strains.

Figure 3b shows the effects of ionic strength on the luminescence of the recombinant *E. coli* with the T3, SP6, or T7 promoter that were induced by 100 μM toluene. The results demonstrated almost no effect of different ionic strengths on the luminescence for SP6-*lux-E. coli*, but the ionic strength had greater effects on that of T7-*lux-E. coli*. When the ionic strength was 0.55 M, the luminescence of T7-*lux-E. coli* decreased by 12.5% ± 0.6%. This inconsistency among the recombinant *E. coli* with different promoters was presumed to be related to promoter structure and composition, which determine the strength of various types of promoter–target DNA bonds [30]. Additional experiments to investigate these differences are planned. In general, the ranges of ionic strengths of groundwater, river water, seawater, and polluted water are 0.01–0.02 M, 10^− 3^–10^− 2^ M, 0.45–0.55 M, and > 10^− 2^ M, respectively. Thus, SP6-lux-*E. coli* is suitable for application in various water environments (groundwater, river water, and seawater), whereas T7-*lux-E. coli* is suitable for use in low ionic strength environments.

### Effects of coexisting carbon sources, intermediates, and toluene analogs on luminescence

**Fig. 4:**
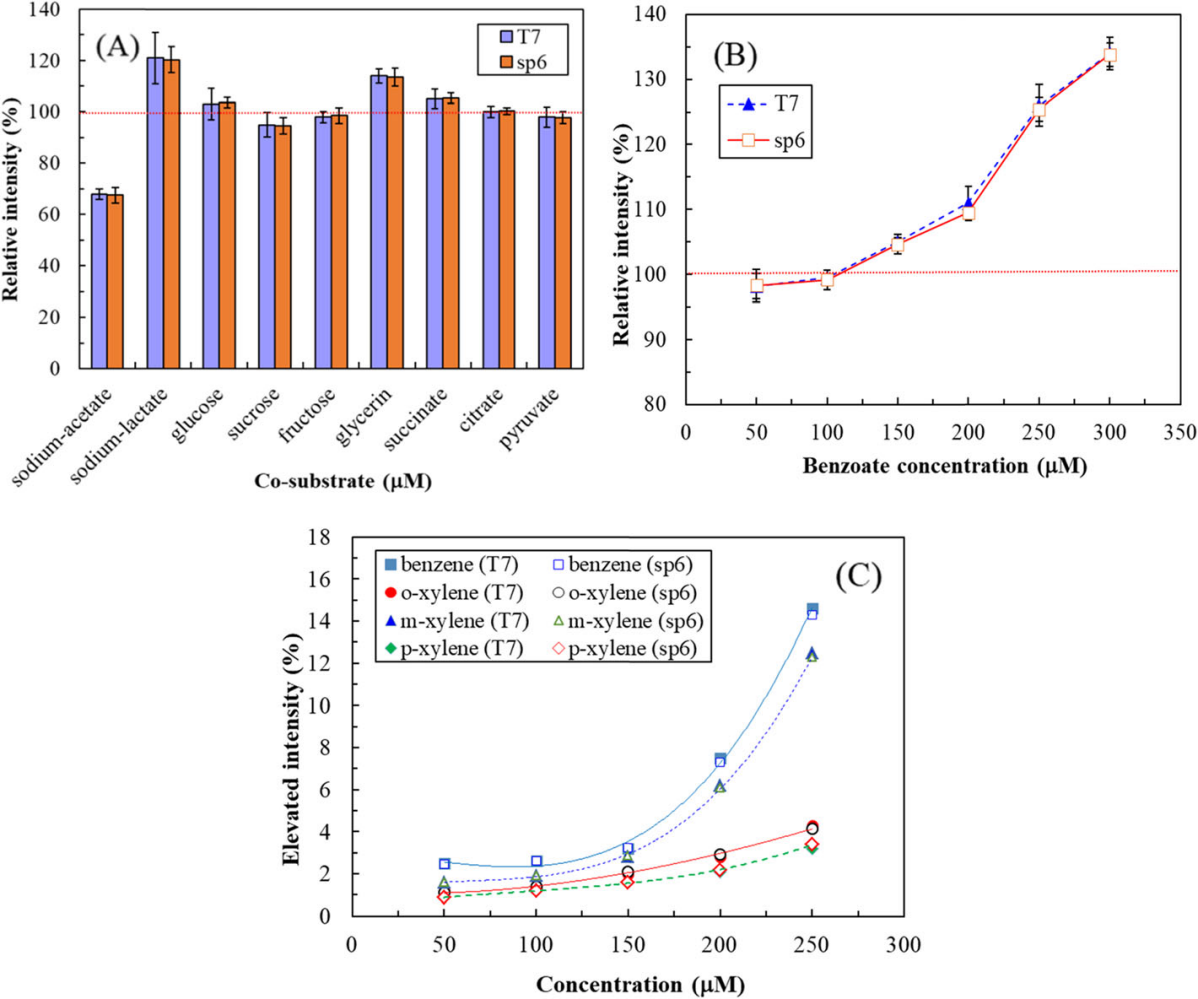
Effects of (**a**) coexisting carbon sources (100 μM), (**b**) benzoate, and (**c**) toluene analogs and their concentrations on luminescence of T7-*lux-E. coli* and SP6-*lux-E. coli* induced by 100 μM toluene for 2 h or 1 h

The *xyl* genes of the *Pseudomonas putida* TOL plasmid encode the genetic information required for the degradation of toluene and related aromatic compounds. The *xylR* and *xylS* genes of the *xyl* structural genes encode the regulatory proteins of the catabolic operons, whereas the XylR protein is the master regulator of TOL plasmid catabolic operons for the metabolism of toluene [31]. Transcription of the operon is positively regulated by the XylR/XylS protein activated by toluene, xylenes, or their alcohol catabolic products [32]. Figure 4a illustrates the effects of coexisting carbon sources at 100 μM on the luminescence of T7-*lux-E. coli* and SP6-*lux-E. coli*. The tested chemicals are considered potential inhibitors or activators (indirect or direct inducers) of *xylS* and *xylR* and may deviate significantly or have an additive effect in relation to theoretically expected effects, calculated on the basis of individual chemicals [12, 33, 34]. The current results demonstrated that the coexistence of lactate or glycerin with toluene induced greater luminescence than did toluene alone. Lactate at 100 μM increased luminescence by 21% ± 8.6% for T7-*lux-E. coli* and 20.3% ± 5.1% for SP6-*lux-E. coli*, while glycerin increased by 14% ± 1.8% for T7-*lux-E. coli* and 13.5% ± 3.5% for SP6-*lux-E. coli*, respectively. The increased luminescence disappeared when the concentrations were below 70 μM (lactate) or 85 μM (glycerin). By contrast, the coexistence of acetate with toluene induced lower luminescence than did toluene alone; luminescence decreased by 32% ± 1.5% for T7-*lux-E. coli* and 32.5% ± 2.9% for SP6-*lux-E. coli*. However, for other chemicals, the coexistence had negligible effect on the detection of toluene by T7-*lux-E. coli* and SP6-*lux-E. coli*.

Figure 4b illustrates the effects of the benzoate concentration on the luminescence of T7-*lux-E. coli* and SP6-*lux-E. coli*. Benzoate is the most important metabolite produced during toluene biodegradation [35], which may affect *XylS* expression [33]; thus, we evaluated the effect of the benzoate concentration on the luminescence of T7-*lux-E. coli* and SP6-*lux-E. coli*. The results demonstrated that a high benzoate concentration could induce higher luminescence than did toluene alone, as detected using T7-*lux-E. coli* and SP6-*lux-E. coli*. Although 50–150 μM benzoate did not affect luminescence, 250–300 μM benzoate increased luminescence by 26% ± 3.5% for T7-*lux-E. coli* (25.4% ± 1.8% for SP6-*lux-E. coli*) and 34% ± 2.8% for T7-*lux-E. coli* (33.8% ± 1.8% for SP6-*lux-E. coli*), respectively. In other words, the effect of low concentrations of benzoate on luminescence was limited when toluene was detected by T7-*lux-E. coli* or SP6-*lux-E. coli*.

Figure 4c illustrates the effects of toluene analogs and their concentrations on the luminescence of T7-*lux-E. coli* and SP6-*lux-E. coli*. The results demonstrated that the various concentrations of *o*-xylene and *p*-xylene had negligible effects on toluene detection by the recombinant *E. coli* biosensor; moreover, even when 250 μM *o*-xylene was used, only 4.15–4.30% increase in luminescence was observed. However, 250 μM *m*-xylene and 250 μM benzene induced T7-*lux-E. coli* or SP6-*lux-E. coli* to produce relatively high luminescence (12.3–12.5% and 14.3–14.6%, respectively). By contrast, the effect of the toluene analog concentration of ≤200 μM on toluene detection was limited (< 8%). The effect of the synergistic mode was far lower than that observed in the *P. putida* mt-2 KG1206 biosensor [12].

Taken together, these results illustrate that our recombinant luminescent biosensor possesses high selectivity and specificity when detecting a group of analytes with similar chemical structures. Because the included chemicals mainly affect the regulatory genes *xylS* or *xylR*, but not the T3, SP6, or T7 promoter, their effects on the magnitude of luminescence among all three recombinant *E. coli* biosensors were similar [12]. Figure 4 exemplifies the cases of T7-*lux-E. coli* and SP6-*lux-E. coli*.

### Relationship of toluene concentration with luminescence

**Fig. 5:**
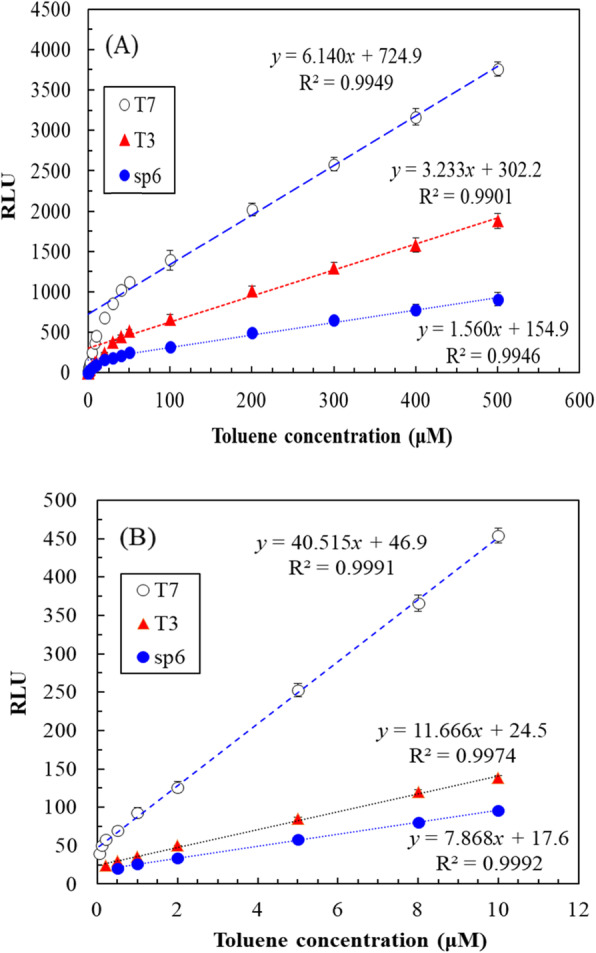
Relationship between toluene concentration [(**a**) 0.01–500 and (**b**) 0.05–10 μM] and luminescence of recombinant *E. coli* with different promoters (initial cell concentration: 5 × 10^7^ cfu/mL, culture media: TMM, operational condition: 37 °C and 200 rpm, incubation time: 2 h for T3/T7-*lux-E. coli* and 1 h for SP6-*lux-E. coli*)

The function of these promoters (T7, T3, SP6) is to make the downstream reporter gene (*lux*) more strongly expressed. Therefore, *xylR* is first induced in the presence of toluene and activates gene expression, then the promoters and reporter gene (*lux*) follow. Under optimal operating conditions, we determined the relationships between the toluene concentration and the luminescence of the three recombinant *E. coli* strains. Two sets of linear relationships were observed between the toluene concentration and luminescence at different concentration ranges. Figure 5a presents a set of regression equations for the toluene concentration and the luminescence of T7-*lux-E. coli*, T3-*lux-E. coli*, and SP6-*lux-E. coli* when the toluene concentration was 10–500 μM: *y* = 6.140*x* + 724.9, *y* = 3.233*x* + 302.2, and *y* = 1.560*x* + 154.9, respectively. Figure 5b presents another set regression equations for T7-*lux-E. coli*, T3-*lux-E. coli*, and SP6-*lux-E. coli* when the toluene concentration was ≤10 μM: *y* = 40.515*x* + 46.9, *y* = 11.666*x* + 24.5, and *y* = 7.868*x* + 17.6, respectively. The coefficients of determination for these equations was high (> 0.99), indicating their reliability. The concentration-dependent differences in these linear relationships may have been due to differences in promoter characteristics [36]. Moreover, for T7-*lux-E. coli*, T3-*lux-E. coli*, and SP6-*lux-E. coli*, the LODs for toluene were 0.05, 0.2, and 0.5 μM, respectively. Therefore, T7*-lux-E. coli* was the most sensitive. Willardson et al. (1998) constructed a bacterial biosensor with the reporter gene *luc*, Casavant et al. (2003) constructed a site-specific recombination-based biosensor with *tbu*A1UBVA2C promoter, Li et al. (2008) constructed a *lux*-based bacterial biosensor, Zeinoddini et al. (2010) constructed a aequorin-based *E. coli* biosensor, Zhong et al. (2011) constructed a monooxygenase biosensor, and Ray et al. (2018) constructed a protein-based biosensor; their LODs for toluene were 10, 0.2, 7.5, 1, 3, and 3.3 μM, respectively [13, 20–22, 37, 38]. Compared with the aforementioned biosystems, T7*-lux-E. coli* has lower LOD (0.05 μM), indicating acceptable sensitivity. To develop a biosensor for detecting toluene, reporter genes such as *luc*, *lux*, and *aequorin* were often constructed downstream of the degradation gene. However, these biosensors could not measure trace levels of toluene contamination in wastewater. To improve the LOD, a promoter (T7, T3, or SP6) was inserted between the degradation gene and reporter gene. To our knowledge, little has been reported on applying this strategy to regulate the expression of the reporter gene and improve the LOD of a biosensor for toluene. In conclusion, the novel plasmid or biosensor with low LOD constructed here exhibited high potential for measuring bioavailable toluene.

Hence, on the basis of the aforementioned reliable equations or the calibration curve for 0.05–10 or 10–500 μM toluene, the toluene concentration in the water samples can be rapidly determined. In addition, the broad detection ranges of T7*-lux-E. coli* indicate that it is a practical toluene measurement tool.

### Reproducibility

To evaluate the reproducibility of the biosensors for detecting toluene, T7-*lux-E. coli*, T3-*lux-E. coli*, and SP6-*lux-E. coli* were tested under identical conditions by using TMM containing 10 μM toluene. Relative standard deviation (RSD) for T7-*lux-E. coli*, T3-*lux-E. coli*, and SP6-*lux-E. coli* was 4.3, 5.1, and 5.8%, respectively (*n* = 10). Batch-to-batch variation was also tested by comparing the luminescence from the five sets, which was tested using TMM containing 10 μM toluene, and the RSD for T7-*lux-E. coli*, T3-*lux-E. coli*, and SP6-*lux-E. coli* was 6.2, 6.5, and 9.4%, respectively. These results are comparable to the reproducibility reported for two induction-based toluene biosensors: RSD = 9.5% for *n* = 3 (with 21.7 μM toluene) and RSD = 7.4% for *n* = 8 (with 92 μM toluene) [38, 39]. Thus, our recombinant luminescent *E. coli* biosensors demonstrated operational stability. Similar results were obtained when these biosensors were applied for measuring 10 μM toluene after a 3-month cryogenic storage period.

### Toluene measurement in groundwater and river water by using our three recombinant luminescent *E. coli* biosensors

Most luminescent biosensors have been applied for measuring toluene availability in artificial wastewater, but few have been applied in actual wastewater. Table 1 summarizes the measured toluene concentrations in seven groundwater samples and three river water samples using our three recombinant luminescent *E. coli* biosensors and the standard GC–MS method. The results demonstrated that the toluene concentration determined using our biosensors and through GC–MS demonstrated excellent correlation (r^2^ > 0.998); moreover, the deviation between the toluene concentrations measured through GC–MS and those measured using T7-*lux-E. coli*, T3-*lux-E. coli*, and SP6-*lux-E. coli* was − 14.3 to 9.1%, − 10.7 to 26.7%, and − 3.6 to 4.2%, respectively. Considering the measurement ranges and accuracy, T7-*lux-E. coli* provided the accurate and reliable toluene measurement in these aqueous matrices. However, under appropriate toluene concentration ranges, SP6-*lux-E. coli* could be the best biosensor in terms of accuracy, and its genetic assembly is relatively less susceptible to environmental interference [26]. The measurement deviation of T7-*lux-E. coli* and SP6-*lux-E. coli* were comparable to that (− 16.7 to 7.5%) of electrochemical inhibition bacterial sensor array for toluene detection [40]. Taken together, these results indicate that the developed recombinant luminescent bacterial biosensors can determine toluene concentration in different water bodies.

**Table 1:** Toluene measurement from groundwater and river water by using the GC–MS method and biosensors

	Groundwater							River water		
GC–MS	0.15^a^	0.56	1.20	9.5	5.6	15.6	0.082	0.12	20.6	31.5
T7-biosensor	0.16 (6.7%)^b^	0.61 (8.9%)	1.31 (9.1%)	8.6 (−9.5%)	4.8 (−14.3%)	15.2 (−2.6%)	0.078 (−4.9%)	0.13 (8.3%)	18.5 (−10.2%)	30.6 (−2.9%)
T3-biosensor	ND^c^	0.50 (−10.7%)	1.52 (26.7%)	10.1 (6.3%)	6.1 (8.9%)	16.5 (5.8%)	ND-	ND-	21.8 (5.8%)	32.6 (3.5%)
SP6-biosensor	ND-	0.54 (−3.6%)	1.25 (4.2%)	9.8 (3.2%)	5.8 (3.6%)	15.9 (1.9%)	ND-	ND-	20.9 (1.5%)	32.1 (1.9%)

^a^Unit: μM

^b^Deviation compared with GC–MS-measured value

^c^ND meaning Not Detected, the value < LOD

## Conclusions

In this study, recombinant luminescent *E. coli* biosensors containing different promoters (T3, T7, and SP6) positioned before the reporter gene *lux* were developed for the accurate measurement of toluene concentrations in groundwater and river water. Of these biosensors, T7*-lux-E. coli* was the most sensitive to toluene, with optimal LOD and widest measurement range for toluene concentrations. Moreover, SP6-*lux-E. coli* had the shortest reaction time and highest adaptability to environmental interference but the poorest LOD. T7*-lux-E. coli* exhibited competitive advantages over previously reported biosystems, particularly for optimal LOD and wide measurement range. According to the results of reproducibility experiments and the test on actual water samples, our *lux-*based biosensors exhibited the high operational stability (i.e., low RSD) and acceptable measurement deviation. In conclusion, our biosensors, particularly T7*-lux-E. coli*, are sensitive, reliable, specific, and stable systems for preliminary in-field detection of toluene in water samples.

## Materials and methods

### Bacterial strains, gene cloning, and biosensor plasmid construction

To clone the *tol* gene, partial *tol* in *P. putida* (ATCC 33015) was amplified using the primer set (forward 5′-GTTAACTGCATCCAGCCC-3′, reverse 5′-CCGGGCGATGCCAACCC-3′) through polymerase chain reaction (PCR). To clone T3-*lux*, SP6-*lux*, or T7-*lux*, *lux* in *Vibrio vulnificus* was amplified with the primer set for the corresponding genes (T7*-lux*, forward 5′- TAATACGACTCACTATAGGTCGACTTTATCGAGCCTGA-3′ and reverse 5′-CAGCTGTTTTTGCTCCT-3′; T3-*lux*, forward 5′- ATTAACCCTCACTAAAGGTCGACTTTATCGAGCCTGA-3′ and reverse 5′-CAGCTGTTTTTGCTCCT-3′; SP6-*lux*, forward 5′-ATTTAGGTGACACTATAGGTCGACTTTATCGAGCCTGA-3′ and reverse 5′-CAGCTGTTTTTGCTCCT-3′) through PCR. All the resultant DNA fragments were inserted into the pET-15b vector plasmid (Promega, Madison, WI, USA). The recombinant plasmids were named pTOL, pT3-*lux*, pSP6-*lux,* and pT7-*lux*. In brief, the plasmids were then transferred to the expression host *E. coli* DH5α and plated on Luria–Bertani (LB) agar plates. Then, isolated pTOL, pT3-*lux*, pSP6-*lux*, and pT7-*lux* plasmids were cut at cleavage sites using *Bst*EII/*Hin*dIII and *Hin*dIII/*San*I. Next, pTOL-T3-*lux*, pTOL-SP6-*lux*, and pTOL-T7-*lux* were constructed by ligating pTOL to pT3-*lux*, pSP6-*lux*, and pT7-*lux* fragments by using T4 DNA ligase (New England BioLabs, Beverly, MA, USA), respectively. The resulting plasmids were inserted into the pET-15b vector plasmid. Next, the plasmids were transformed into *E. coli* DH5α to create the corresponding whole-cell biosensors. All the restriction enzymes were purchased from New England BioLabs. Vector DNA was prepared using the QIAEX II gel extraction kit (Qiagen, Hilden, Germany).

### Bacterial growth

*E. coli* with pTOL-T3-*lux* (T3-*lux-E. coli*), pTOL-SP6-*lux* (SP6-*lux-E. coli*), and pTOL-T7-*lux* (T7-*lux-E. coli*) (all initial concentration = 2 × 10^5^ cfu/mL) were cultivated in LB broth containing 50 mg/L ampicillin at 37 °C at 200 rpm on an orbital shaker. Overnight cultures were then diluted 100-fold into toluene-mineral medium (TMM) containing 0.43 g/L K_2_HPO_4_, 0.23 g/L KH_2_PO_4_, 1 g/L NH_4_NO_3_, 0.2 g/L MgSO_4_^.^7H_2_O, 0.1 g/L CaCl_2_, 0.05 mg/L Fe_2_(SO_4_)_3_, 0.25 mg/L NaMoO_4_^.^2H_2_O, 50 mg/L ampicillin, and a specific concentration of toluene (in this case: 10 mg/L). The cultures were incubated at 37 °C at 200 rpm on an orbital shaker. The optical density (OD) measurements of the bacterial growth and the luminescence intensity released from recombinant *E. coli* were conducted at specific intervals. The OD of the cultures was measured at 600 nm on a UV-vis spectrophotometer (Shimadzu, Kyoto, Japan). The luminescence intensity [in relative light units (RLU)] was measured by adding 200 μL of the culture to a 96-well microplate and then placing it under a microplate luminometer (Titertek-Berthold, Pforzheim, Germany). All chemicals used in the experiment were of analytical grade (purity > 99%). Toluene was purchased from Sigma-Aldrich Corporation (St. Louis, MI, USA).

### Determination of optimum conditions

After 12 h of cultivation in TMM, 1 mL of culture containing 5 × 10^7^ cfu/mL T3-*lux-E. coli*, SP6-*lux-E. coli*, or T7-*lux-E. coli* was inoculated into 200 mL of TMM [with different final concentrations (50–500 μM) of toluene] and incubated at 37 °C on an orbital shaker (200 rpm) for 5 h. The luminescence intensity was continuously measured until the luminescence intensity approached zero. The effects of temperature and ionic strength on bioluminescence emissions of the three recombinant *E. coli* strains were evaluated separately, and 100 μM toluene was used as an inducer in TMM. During incubation, temperature (15–40 °C) was controlled using thermostat, and ionic strength (0.04–0.55 M) was adjusted using aqueous NaCl. After 2-h incubation for T3-*lux-E. coli* and T7-*lux-E. coli* and 1-h incubation for SP6-*lux-E. coli*, 200 μL of the cultures were sampled, and the luminescence intensity (in RLU) of these biosensors was measured immediately. On average, 20-min consecutive measurements were recorded (i.e., one measurement every 0.5 s).

Various carbon sources (i.e., acetate, lactate, glucose, sucrose, fructose, glycerin, succinate, citrate, and pyruvate) were added to TMM to evaluate their effects on bioluminescence emissions of the three recombinant *E. coli* strains. In medium, final concentrations of coexisting carbon sources and toluene were 100 μM. After 2-h incubation for T3-*lux-E. coli* and T7-*lux-E. coli* and 1-h incubation for SP6-*lux-E. coli*, the luminescence intensity of each biosensor was measured immediately. Toluene analogs (i.e., benzene, *o*-xylene, *p*-xylene, and *m*-xylene) and intermediates of toluene degradation (benzoate) were added to TMM to evaluate the effects on the bioluminescence emissions of the three recombinant *E. coli* strains. Based on their solubility, *o*-xylene, *p*-xylene, and *m*-xylene were predissolved in 95% ethanol and added to TMM. The final concentrations of the toluene analogs, benzoate, and toluene in medium were 50–250, 50–300, and 100 μM, respectively. The cells were incubated for 2 h (T3-*lux-E. coli* and T7-*lux-E. coli*) or 1 h (SP6-*lux-E. coli*) at 37 °C; the luminescence intensity (in RLU) of these biosensors was then measured, as described above. Measurements were obtained from at least three independent experiments, each performed at least in triplicate.

### Establishment of calibration curve and measurement of real water sample

To establish the relationships between the toluene concentration and the luminescence intensity of the three recombinant *E. coli* biosensors, we mixed 100 μL of toluene (0.01–500 μM), 50 μL of 4× TMM (without toluene), and 50 μL of recombinant luminescent *E. coli* cells (final concentration after mixing: 5 × 10^7^ cfu/mL). We then operated at the optimal incubation time and conditions determined in previous experiments. Standard curves (known as calibration curves) were plotted from the linear regression of average luminescence intensity at each toluene concentration. To obtain the LOD concentration, we calculated the SD from the average of the three blank measurements, multiplied the SD by 3, and then used the standard curve to determine the LOD concentration. To ensure that the established curves and methods were valid, we prepared similar solutions as mentioned above, but used groundwater (from Lin-Yuan Industrial Park, Kaohsiung City, Taiwan) and river water (from Tamsui River, New Taipei City, Taiwan) instead of pure toluene. The toluene concentration in the prepared solution was separately measured using the established GC–mass spectrometry (MS) method [41] as well as using our three recombinant *E. coli* biosensors. Considering practical application, the retention of illuminance of recombinant *E. coli* after its cryogenic storage is essential for biosensor usage; thus, similar experiments were conducted when the biosensors were cryogenically stored for 3 months. Data were obtained from at least three independent experiments, with each performed at least in triplicate.

## Data Availability

All data generated or analyzed during this study are included in this published article.
